# Patterned Metal/Polymer Strain Sensor with Good Flexibility, Mechanical Stability and Repeatability for Human Motion Detection

**DOI:** 10.3390/mi10070472

**Published:** 2019-07-15

**Authors:** Xu Zheng, Qing Wang, Jinjin Luan, Yao Li, Ning Wang

**Affiliations:** Institue of NanoEngineering, College of Civil Engineering and Architecture, Shandong University of Science and Technology, Qingdao 266590, Shandong, China

**Keywords:** patterned strain sensor, mechanical property, repeatability, flexible electronics, human motion detection

## Abstract

Wearable health monitoring smart systems based on flexible metal films are considered to be the next generation of devices for remote medical practice. However, cracks on the metallic surface of the films and difficulty in repeatability are the key issues that restrict the application of such wearable strain sensors. In this work, a flexible wearable strain sensor with high sensitivity and good repeatability was fabricated based on a patterned metal/polymer composite material fabricated through nanoimprint lithography. The mechanical properties were measured through cyclic tension and bending loading. The sensor exhibited a small Δ*R/R*_0_ error line for multiple test pieces, indicating the good mechanical stability and repeatability of the fabricated device. Moreover, the sensor possesses high sensitivity with gauge factors of 10 for strain less than 50% and 40 for strain from 50% to 70%. Various activities were successfully detected in real-time, such as swallowing, closing/opening of the mouth, and multi-angle bending of elbow, which illustrates the proposed sensor’s potential as a wearable device for the human body.

## 1. Introduction

Wearable strain sensors attached to the human body have been explored recently as key components for potential applications in human motion detection and personal healthcare devices [1,2,3]. Flexibility, stretchability, good sensitivity, and conductivity are the essential characteristics of such smart wearable electronics to respond to the varied strains caused by human activities [4,5,6,7]. Great progress has been made in fabricating wearable strain sensors using metallic and carbon nanomaterials on flexible polymer to achieve these properties [8,9,10,11,12]. However, under external load conditions, cracks on the metallic surface and difficulty in repeatability are the key issues that restrict the application of wearable strain sensors [13,14].

Among various wearable strain sensors, nano-metal films were frequently applied as the sensitive component in the sensor due to their excellent electrical conductivity and ductility [15]. However, external stresses can easily lead to the initiation of cracking in the surface of metal films, which results in a loss of durability, stability, and sensitivity for the device [16,17]. To solve the problem of circuit breaking caused by metal cracks, metal nanowires spin-coated on the polymer substrates were used instead of a deposited metal film [18,19]. The metal nanowires could be connected together as elastic conductive bridges to maintain conductivity because of the high length–diameter ratio. Still, the metal nanowire film could fail under large strain conditions due to the disconnection of the metal wires [20]. Another method was proposed in which metal films were deposited on the wrinkled substrates via pre-stretching, solvent treatment, and heat induction, which could successfully reduce the cracks on the metal surface under large deformation conditions [21,22,23,24]. However, the materials of nanowires and wrinkled patterns are irregular and difficult to be reproduced, resulting in the need for the performance detection and calibration of each device before use, thereby illustrating that these materials are unsuitable as wearable strain sensors. More importantly, because of the instability and randomness of the strain sensor in these investigations, no repeated experiment has been reported, which has made it difficult to apply the devices prepared in the research to industrial production. The objectives of this research are to enhance the mechanical stability and repeatability of a wearable strain sensor based on a regular patterned composite material.

In this paper, a wearable strain sensor with regular periodic surface patterns is fabricated with the aim of improving the stability and repeatability of the sensor through nanoimprint lithography (NIL). The mechanical stability was investigated by measuring the surface resistance change (Δ*R/R*_0_) for initial state and cyclic loads. These experiments were explored on multiple repetitive tests to investigate repeatability. Then, the patterned wearable strain sensor was evaluated via attaching it to a human body to detect various activities.

## 2. Experimental Section

### 2.1. Materials and Characterization

Polydimethylesiloxane (PDMS) precursor and PDMS curing agent were purchased from Dow Corning SYLGARD (Midland, MI, USA). The purity of the silver target used for deposition was 99.99%. The SiO_2_ mold (25 × 25 mm^2^) had a periodic line array patterned with pitch of 200 nm, line width of 100 nm, and depth of 200 nm. Although it is more conducive to improving the mechanical stability of patterned devices with a small period and large aspect ratio, the imprinting and demolding process with a high aspect ratio and small period is a major issue in nanoimprinting technology [25,26,27]. The period of 200 nm and aspect ratio of 2:1 are relatively mature parameters in nanoimprint technology, being more conducive to the successful preparation of patterns.

A vacuum drying oven (Lange, IPC-25, Baoding, China) was employed to cure the PDMS polymer. The metal layer was deposited by vacuum thermal evaporation (Vnano, VZZ-300, Beijing, China). Scanning electron microscopy (SEM; Hitachi, S-4800, Tokyo, Japan) images were obtained to study the morphology features of the films. The resistances of the strain sensor were measured using a multimeter (UNI-T, UT890C+, Dongguan, China).

### 2.2. Device Fabrication

**Figure 1:**
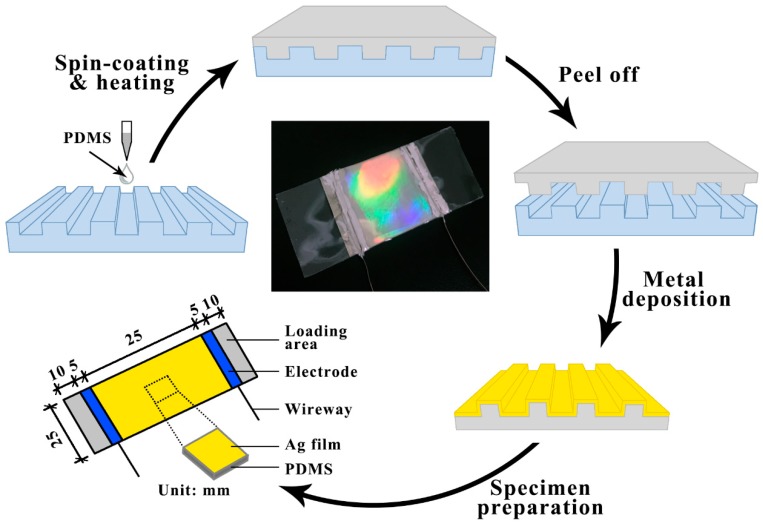
Schematic diagram of the fabrication process of the patterned strain sensor specimen.

Figure 1 shows a schematic diagram of the fabrication of the patterned metal/polymer device for a wearable strain sensor through NIL. The elastic layer PDMS (weight ratio of 10:1) was spin-coated on the patterned mold, and then the PDMS was cured under a pressure of 25 bar at 70 °C for 4 h in the vacuum drying oven [28]. After being peeled off the mold, Ag thin film (100 nm thickness) was deposited on the patterned PDMS substrate. A specific deposition condition (deposition current = 170 A) and cooling condition (PDMS substrate was fixed on the cooling plate) were employed to fabricate the specimens. Then, two copper wires were connected to the Ag film using an electrode (silver paste), and the other ends of the copper wires were connected to the multimeter. Both ends of the specimen were the loading regions. Finally, a PDMS film was spin-coated and cured on the surface of the patterned film to prevent the Ag layer from being oxidized, leading to interface failure. The size of the fabricated sensor is shown in Figure 1. The center of Figure 1 shows the picture of the patterned PDMS/Ag/PDMS strain sensor specimen.

## 3. Results and Discussion

### 3.1. Morphology of the Patterned Strain Sensor

**Figure 2:**
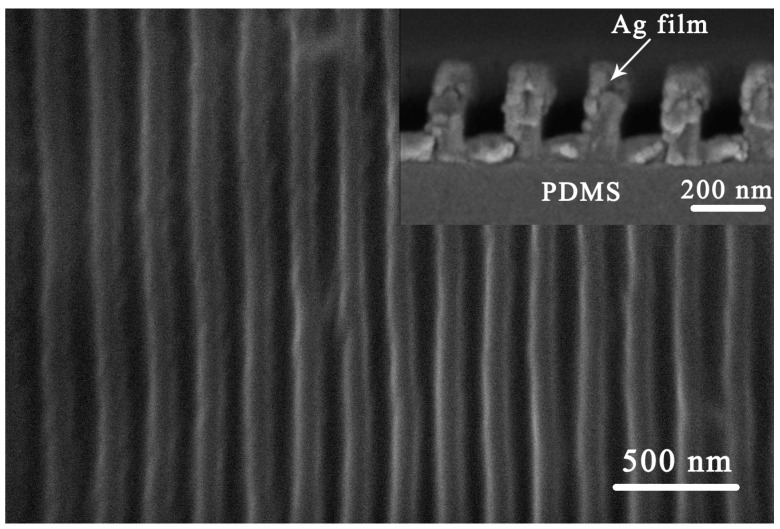
The SEM images of the patterned strain sensor.

Figure 2 shows the SEM images of the patterned strain sensor with nanoscale grating patterns. The top-view SEM image of the patterned strain sensor in Figure 2 clearly shows the grating structure with a period of 200 nm. The sectional view shows that the mold is completely filled and the Ag film has a thickness of 100 nm on the horizontal surfaces. The Ag film on the side walls or corners of the patterned PDMS surfaces is weaker than the horizontal surfaces due to the vertical deposition of the Ag film during the vacuum thermal evaporation process. The results show that the mold patterns were perfectly transferred to the film surface through reversal imprint, and the Ag film covered the PDMS surface.

### 3.2. Mechanical Stability

#### 3.2.1. Tensile Property and Gauge Factor

**Figure 3:**
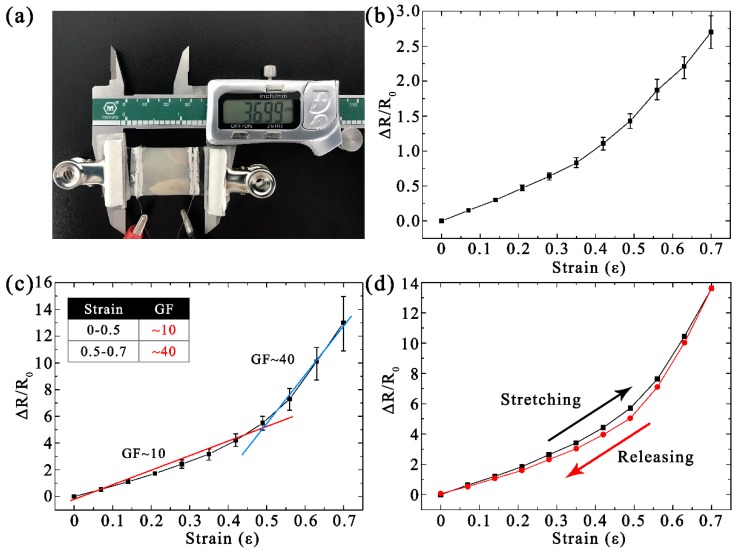
(**a**) Photograph of the experimental setup for measuring the resistance under different amounts of strain. The measured strain-dependent relative Δ*R/R*_0_ of the patterned strain sensor under (**b**) the initial state and (**c**) after cyclic loading. (**d**) Hysteresis curves under stretch/release cycles and 0.7 applied strain.

Figure 3a shows the setup used to measure the resistance for the sensor under different amounts of strain. The sensor was secured to a Vernier caliper by two insulating jigs. The appropriate strain was achieved by moving the Vernier caliper. The resistance was measured by the multimeter, which was connected to the alligator clip and the copper wires. Figure 3b,c shows the measured Δ*R/R*_0_ of the patterned strain sensor under different amounts of strain to investigate the tensile properties. The values in Figure 3b,c are the averages of six samples, and the error lines are the maximum and minimum values from the six measured samples employed to explore the stability and repeatability. Figure 3b shows the Δ*R/R*_0_ of the patterned strain sensor under the initial state. The initial average resistance (*R*_0_) of the strain sensor was 14.3 Ω, which is higher compared to the previous studies for bare Ag/PDMS composite material. This is because the Ag films on the side walls or corners of the patterned PDMS surfaces are weaker than the horizontal surfaces. In addition, the Δ*R/R*_0_ of the specimens increased with the strain. The Ag film on the patterned PDMS had a small Δ*R/R*_0_ and a small error range, indicating that the fabricated strain sensor has good stability and repeatability. This is more significant for cyclic loading.

Figure 3c shows the Δ*R/R*_0_ of the patterned strain sensor after cyclic loading (1000 cycles with a strain of 0.5). The *R*_0_ change rate of the strain sensor was approximately 72% (from 14.3 to 24.6 Ω) after cyclic loading. More importantly, when the strain was below 0.5, the Δ*R/R*_0_ error lines of the strain sensor were very small, indicating that the patterned films have good stability under a cyclic tensile load for six repetitive tests. As the strain continued to increase, the Δ*R/R*_0_ error lines gradually expanded. With the stretching of the patterned sensor, this process can be divided into two stages: film expansion and crack propagation. When the strain increases, the patterned metal film will be spread with the stretched substrate, which is still a continuous metal surface. There are few cracks at this stage, so the error lines of this stage will be very small. As the strain continues to increase, the Ag film will produce small cracks in the weakened place.

The gauge factor (GF, GF = (Δ*R/R*_0_)/*ε*), calculated by the slope of the colored curve in Figure 3c, can be used to evaluate the sensitivity of the strain sensor [29,30]. The strain of the sensor increased to 50%, showing a moderate GF of ~10. The GF significantly increased to ~40 with the strain range from 0.5 to 0.7. The increase in the GF of the sensor is attributed to the small cracks in the weak points caused by the large strain. The patterned strain sensor possessed not only a higher GF but also a higher sensing range, which is attributed to the periodic nanopatterns on the surface [5,31].

Figure 3d shows the hysteresis curves under stretch/release cycles and 0.7 applied strain. The specimen used is patterned sensor after cyclic loading. When the strain was reduced from 0.7 to 0, only a slight hysteresis was observed with the ∆*R/R*_0_ increased by 6.2% relative to the initial value owing to the cracks in the Ag film. These results indicate that the patterned strain sensor is highly reversible with minimal hysteresis during the process of stretching/releasing within a strain of 0–0.7 [6,32].

#### 3.2.2. Bending Property

**Figure 4:**
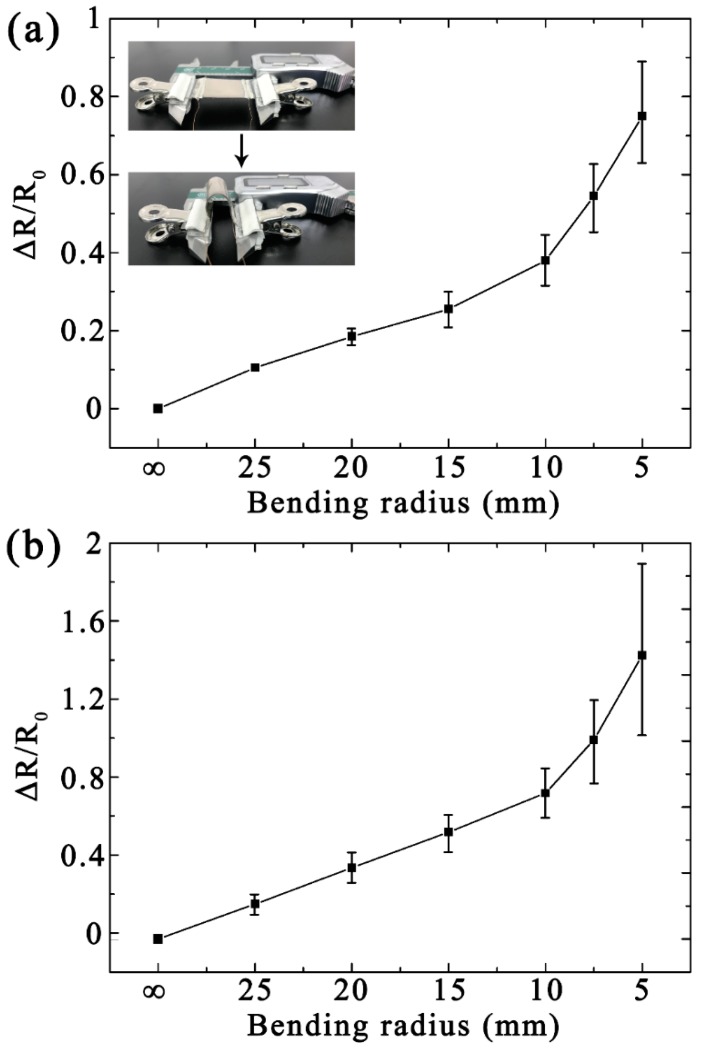
Measured bending-dependent relative Δ*R/R*_0_ of the patterned strain sensors under (**a**) the initial state and (**b**) after cyclic loading.

Figure 4 shows the bending properties of the patterned strain sensor. Similar to the tensile test, the sensor was secured to a Vernier caliper by two insulating jigs, and the appropriate bending radius was achieved by moving the Vernier caliper. The average *R*_0_ was 14.3 Ω, as mentioned above, under the initial state. As shown in Figure 4a, the Δ*R/R*_0_ of the specimens increased with the bending radius. According to the error lines shown in Figure 4a, the device maintained stable performance with a bend radius of less than 10 mm. Figure 4b shows the Δ*R/R*_0_ of the patterned strain sensor after cyclic loading (1000 cycles with a bending radius of 10 mm). The average *R*_0_ change rate of the strain sensor was approximately 22% (from 14.3 to 17.5 Ω) after cyclic loading. At this point, this strain sensor exhibited good bending resistance. It can be seen that from the error lines of Figure 4b that the Ag film on the patterned PDMS substrate still had good stability when the bending radius reached 10 mm. The good bending resistance and stability can be mainly attributed to the obviously improved mechanical occlusion effect induced by the contact properties of the patterned PDMS surface.

### 3.3. Human Motion Monitoring

**Figure 5:**
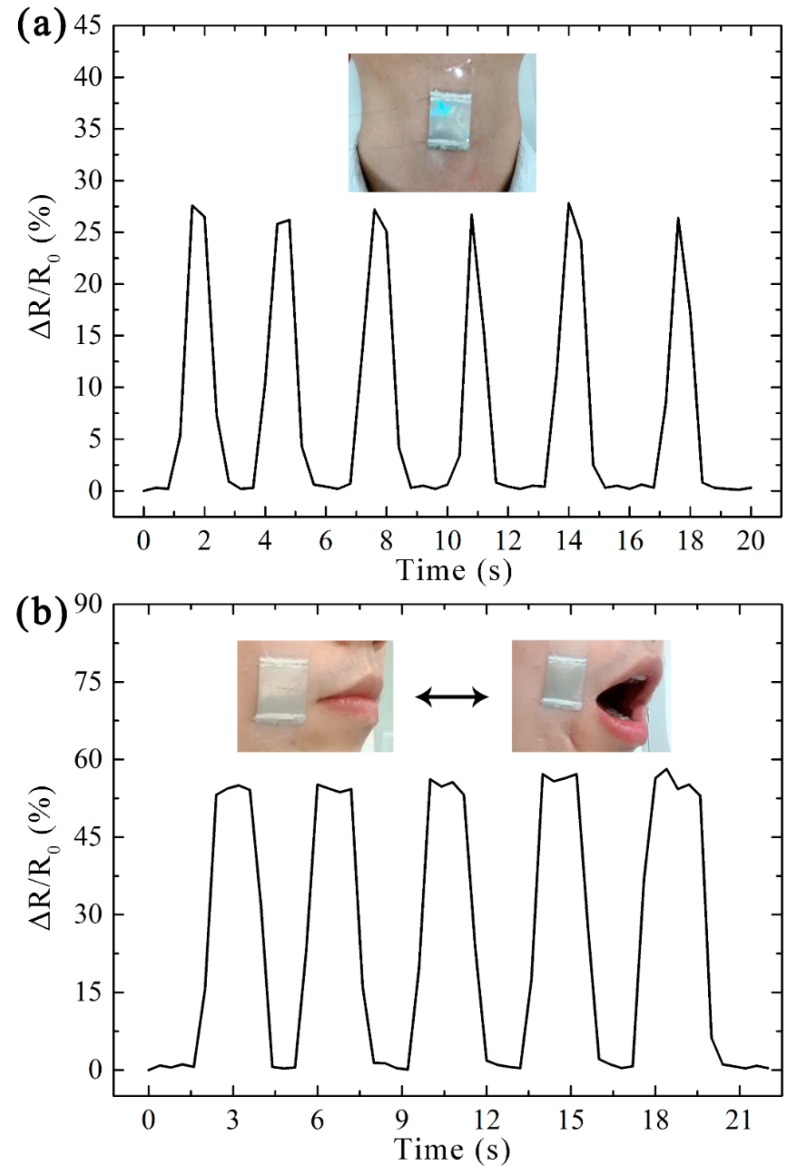
Photographs and real-time Δ*R*/*R*_0_ response of the strain sensor attached to (**a**) the neck and (**b**) the mouth to detect small activities.

In order to ensure the stability of the strain sensor, the specimen used for human motion monitoring was measured after cyclic stretching. The excellent sensitivity of the patterned sensor indicates that it can be applied to monitor small activities. Figure 5a shows the process of monitoring swallowing activities through the direct attachment of the sensor on the neck. The activities of the throat cause obvious movement when swallowing. As a result, the measured values of Δ*R/R*_0_ reached up to 27% for swallowing when the strain sensor was placed on the neck. In addition, when the strain sensor was attached to muscles around the mouth, and the observed Δ*R/R*_0_ was approximately 55% responding to the opening and closing process of the mouth, as shown in Figure 5b.

**Figure 6:**
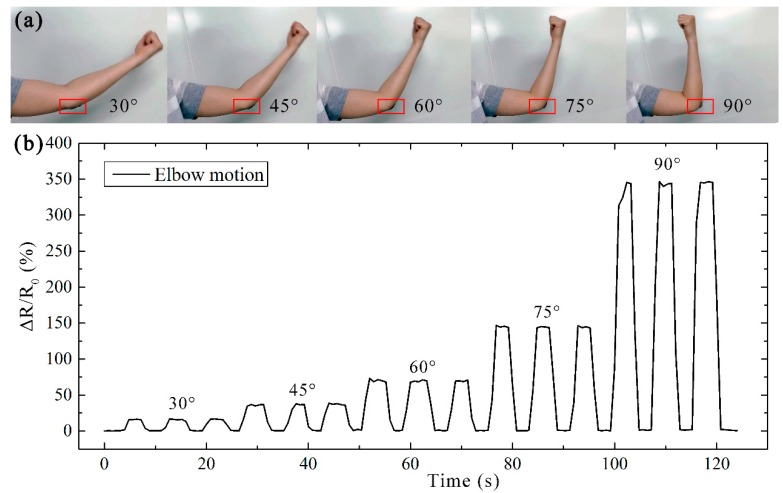
Elbow motion detection based on the patterned strain sensor. (**a**) Photographs of a real device under various bending angles, including 30°, 45°, 60°, 75°, and 90°. (**b**) Real-time Δ*R*/*R*_0_ responses of the patterned strain sensor to cyclic motions of elbow bending.

Based on its good sensitivity and stability, the patterned strain sensor can also be applied to detect human motions in large activities. Figure 6 shows the monitoring of elbow bending. To better demonstrate the performance of the fabricated sensor, as shown in Figure 6a, the strain sensor was employed to detect the multi-angle bending of the elbow, at 30°, 45°, 60°, 75°, and 90°. As the bending angle of the arm increased, the strain sensor responses via a real-time signal showed increased values of Δ*R*/*R*_0_, as shown in Figure 6b. Moreover, for the frequently varied bending angles, the strain sensor showed excellent response and quick relaxation.

As shown in Figure 5 and Figure 6, the varied activities can be distinguished according to the peaks of the output signal. More importantly, due to the good reversibility of the sensor, the resistance can almost return to its initial value for each cycle during varied activities. Based on the intensity of the applied external load, the sensitive response proves the great abilities of our sensor for the detection and quantification of strain. These results illustrate that the proposed patterned strain sensor employed as a wearable device is capable of monitoring various activities of the human body in real time.

## 4. Conclusions

A wearable strain sensor with high sensitivity and good repeatability based on a patterned Ag/PDMS composite material was successfully fabricated by NIL. The preparation method used in this paper resulted in a sensor that possesses good stability and repeatability under cyclic tensile or bending loading for repeated experiments. Moreover, the patterned strain sensor exhibited high sensitivity with GFs of 10 at a strain of less than 50% and 40 at a strain in the range of 50 to 70%. Based on such desirable features, this sensor was successfully applied for the real-time detection of human motion activities from subtle activities to large joint motions. The varied activities could be clearly distinguished according to the peaks of the output signal. Moreover, owing to the excellent reversibility of the sensor, the resistance can almost return to its initial value for each cycle during the various activities. The good mechanical flexibility, stability, and repeatability of the patterned strain sensor illustrate its potential as a wearable device for the detection of various activities of the human body.
